# Association of rs2910164 in miR-146a with type 2 diabetes mellitus: A case–control and meta-analysis study

**DOI:** 10.3389/fendo.2022.961635

**Published:** 2022-09-27

**Authors:** Wei-Wei Chang, Li-Ying Wen, Liu Zhang, Xin Tong, Yue-Long Jin, Gui-Mei Chen

**Affiliations:** ^1^ Department of Epidemiology and Health statistics, School of Public Health, Wannan Medical College, Wuhu, China; ^2^ Department of Hospital Infection Management Office, Wuhu Hospital of Traditional Chinese Medicine, Wuhu, China; ^3^ School of Health management, Anhui Medical University, Hefei, China

**Keywords:** mir-146a, type 2 diabetes mellitus, meta-analysis, case-control, polymorphism

## Abstract

**Objective:**

Several studies have shown that miR-146a rs2910164 (C > G) is associated with type 2 diabetes mellitus (T2DM) susceptibility, but the results are still controversial. This study is divided into two parts, and one is to explore the relationship between miR-146a rs2910164 polymorphism and the genetic susceptibility of T2DM in Chinese Han population. Second, a meta-analysis on the basis of a larger sample size was used to determine whether this is a susceptibility gene for T2DM.

**Methods:**

A case–control study including 574 T2DM patients and 596 controls was used to evaluate the association of miR-146a rs2910164 polymorphism with the risk of T2DM in Chinese Han People. Then, we systematically searched studies investigating the correlation between miR-146a rs2910164 polymorphism and T2DM susceptibility published before April 2022 from PubMed, Web of Science, Wanfang, and China National Knowledge Infrastructure database, and a meta-analysis including six studies was carried out. The results were expressed by odds ratio (*OR*) and its 95% confidence interval (95% CI).

**Results:**

In a case–control study, we found that there were no statistical differences in genotype frequencies between T2DM and control group. Subgroup analysis showed that, compared with the CC genotype, CG + GG genotype was associated with a decreased risk of T2DM in the subgroup of individuals ≥ 65 years old (*OR* = 0.75; 95% CI: 0.58–0.98; *P*_adjusted_ = 0.032) and BMI < 18.5 (*OR* = 0.16; 95% CI: 0.03–0.89; *P*_adjusted_ = 0.037). In overall meta-analysis, significant heterogeneity was detected. No significant association between miR-146a rs2910164 polymorphism and T2DM was observed in all genetic models under random effects models. Subgroup analysis revealed that there was a significant difference in genotype frequencies between the T2DM and control group in recessive model (CC vs. CG + GG: *OR* = 1.79; 95% CI: 1.08–2.96; *P_Q_* = 0.307, *I* ^2^ = 4.0%) and homozygote model (CC vs. GG: *OR* = 1.79; 95% CI: 1.07–3.00; *P_Q_* = 0.216, *I* ^2^ = 34.7%) in Caucasians.

**Conclusion:**

The results of our study demonstrate that the miR-146a rs2910164 polymorphism might have ethnicity-dependent effects in T2DM and may be related to T2DM susceptibility in Caucasians.

## Background

Type 2 diabetes mellitus (T2DM) is a metabolic disorder characterized by hyperglycemia, which is caused by both insulin resistance and a β-cell deficiency (1). The morbidity and mortality of T2DM are increasing worldwide; it is a major cause of death and disability, after cardiovascular disease and cancer (1). It was estimated that the prevalence of DM in 2019 was 9.3%, affecting approximately 463 million people (with T2DM accounting for 90% of cases), and the prevalence is predicted to increase to 10.9% by 2045 (2). The causes of T2DM are complex, including long-term interactions between environmental and genetic factors (3, 4). A genome-wide association study has identified more than 400 gene loci that are closely related to an increased risk of T2DM (5). Therefore, in addition to environmental factors (age, family history, obesity history, diet, etc.), genetic variation is an important risk factor for T2DM.

Single nucleotide polymorphisms (SNPs) are the most common form of genetic variation in humans. These polymorphisms can alter gene expression and function and are used to predict the risk of disease and clinical prognosis (6). MicroRNAs (miRNAs), which contribute to epigenetic regulation, are small non-coding single-stranded RNAs composed of approximately 22 nucleotides (7). There are approximately 45,000 miRNAs targeting sites in the human genome expected to influence gene expression (7). Various studies have indicated that miRNAs played important roles in glucose metabolism and insulin synthesis (8–11). SNPs in miRNAs can affect the expression of mature miRNAs and the regulation of target genes involved in the occurrence and development of various diseases. The effects of miRNA SNPs on diseases have become a major concern in recent years (12–14).

Rs2910164, located in the seed region of miR-146a, could promote the expression of miR-146a, resulting in immune suppression (15–17). Animal experiments and population studies have shown that the abnormal expression of miR-146a is related to T2DM (18–20). In particular, SNPs in miRNAs might be related to the susceptibility and development of T2DM. Several case–control studies have investigated the correlation between rs2910164 and the risk of T2DM (21–26); however, these studies have yielded inconsistent results. The aim of the current study was to systematically explore the correlation between the rs2910164 polymorphism and T2DM. First, using a large sample case–control design, we validated the effect of the rs2910164 polymorphism on T2DM susceptibility in the Chinese Han population. Second, we conducted a meta-analysis of published studies exploring the correlation between rs2910164 polymorphism and T2DM susceptibility in the general population, providing an experimental basis for the genetic diagnosis of T2DM.

## Materials and methods

### Case–control study

#### Sample size calculation and study population

We selected the miR-146a rs2910164 C > G polymorphism accordingly to verify its association with T2DM, and the SNP is significantly associated with various diseases (27–30). The participants were recruited from individuals undergoing a routine physical examination in Anhui Province (Liuan City and Fuyang City). According to the inclusion and exclusion criteria, a total of 574 unrelated patients with T2DM were assigned the T2DM group and 596 individuals without T2DM patients were assigned the control group. Inclusion criteria for the T2DM group were as follows: (1) age ≥ 55 years, (2) a fasting plasma glucose (FPG) ≥ 7.0 mmol/L or a 2-h post-glucose level ≥ 11.1 mmol/L or a previous diagnosis of T2DM, (3) no family history of DM, (4) Han nationality, and (5) no familial relationship with other patients in the T2DM group. Inclusion criteria for the control group were as follows: (1) age ≥ 55 years, (2) FPG < 6.1 mmol/L and OGTT 2-h PG < 7.8 mmol/L, (3) no family history of DM, and (4) Han nationality. Exclusion criteria for the two groups were as follows: (1) severe consumptive diseases, including viral infection, hyperthyroidism, and malignant tumor and so on; (2) those who are unwilling to sign informed consent and complete the questionnaire; and (3) the inspection items required for the study are incomplete.

#### Data collection

All participants were investigated by questionnaire, physical examination, and laboratory test. The participants were interviewed face to face according to the content of the questionnaire, and the questionnaire was filled out by the respondents. The contents of the questionnaire included gender, age, and history of diabetes, hepatitis, kidney disease, malignant tumor, and cardiovascular disease. Physical examination included height (m), body weight (kg), waist circumference (cm), and hip circumference (cm). BMI was calculated based on the height and weight and using the formula BMI = weight (kg)/height^2^ (m^2^). According to the criteria, subjects were divided into three groups: underweight (< 18.5 kg/m^2^), normal weight (18.5–24 kg/m^2^), and overweight and obesity (≥ 24 kg/m^2^) (31). The waist-to-hip ratio (WHR) was calculated as follows: WHR = waist circumference (cm)/hip circumference (cm). After fasting for 8 h, 2 ml of fasting venous blood was collected to measure FPG.

#### DNA extraction and genotyping

After obtaining informed consent from the participants, 5 ml of peripheral venous blood was drawn from T2DM patients and controls with EDTA anticoagulation tubes. Whole blood genomic DNA was extracted from each participant using according to manufacturer’s instructions of DNA Extraction Kit (QIAGEN FlexGene^®^, Dusseldorf, Germany) and placed in a refrigerator at -80°C. The DNA quality was assessed by Horizon^®^ 58 Agarose Gel Horizontal Electrophoresis (Biometra Corporation, Gottingen, Germany), and the DNA concentration was measured by NanoDrop-2000 Micro Volume UV Spectrophotometer from NanoDrop Corporation (Waltham, MA, USA). MiR-146a rs2910164 SNP genotyping work was performed using a custom-by-design 48-Plex SNPscan™ Kit (Cat#: G0104; Genesky Biotechnologies Inc., Shanghai, China). This kit was developed according to patented SNP genotyping technology by Genesky Biotechnologies Inc., which was based on double ligation and multiplex fluorescence polymerase chain reaction (PCR). Briefly, 100–200 ng of DNA sample was first denatured at 98°C for 5 min in a 10-ml reaction containing 1× DNA lysis buffer and then mixed well with a 10-ml ligation premix composed of 2 ml of 103 ligase buffer, 0.5 of ml ligase, 1 ml of probe mix, and 7.5 of ml Milli-Q water. The ligation reaction was carried out in an ABI2720 thermal cycler. Forty-eight plex fluorescence PCR reactions were performed for each ligation product. PCR reactions were prepared in a 20-ml mixture containing 1× PCR master mix, 1 ml of primer mix set A or set B, and 1 ml of ligation product. PCR products were separated and detected by capillary electrophoresis in an ABI3730XL sequencer. Raw data were analyzed according to the information obtained for the labeling dye color and fragment size of the allele-specific ligation-PCR product. The probe sequences of miR-146a rs2910164 SNP are shown as follow: forward 5’-ATGGGTTGTGTCAGTGTCAGACATS-3’ and reverse 5’-TGAAATTCAGTTCTTCAGCTGGGA-3’. Genotyping was conducted without any knowledge regarding the subject’s case or control status. For quality control, repeated analyses were accomplished to guarantee the genotyping quality by randomly choosing 3% of samples with high DNA quality.

#### Statistical analysis

All statistical analyses were performed using SPSS 25.0 software. The Kolmogorove–Smirnov test was performed to assess the normality distribution of the quantitative data. The quantitative data that conform to a normal distribution are expressed by the mean and standard deviation (SD), and the quantitative data that do not fit a normal distribution are expressed by the median (*P*_25_, *P*_75_). The qualitative data were described by rate or composition ratio. A chi-square (*χ* ^2^) test was used to calculate whether the genotype distribution of T2DM group and normal control group were consistent with Hardy–Weinberg equilibrium (HWE). The association between different genotypes and the risk of T2DM was analyzed using logistic regression, and the results were expressed by odds ratio (*OR*) and its 95% confidence interval (95% CI) and then stratified by age, gender, and BMI. *P* < 0.05 on both sides was considered as statistically significant.

### Meta-analysis

#### Literature search strategy

The present meta-analysis followed the Preferred Reporting Items for Systematic Reviews and Meta-Analyses (PRISMA) guidelines (32). We systematically searched literature published before April 2022 from PubMed, Web of Science, Wanfang, and China National Knowledge Infrastructure (CNKI) database, respectively. The English search terms for the PubMed and Web of Science searches are as follows: (“diabetes mellitus” or “DM” or “diabetes”) and (“miR-146a” or “microRNA-146a” or “miRNA-146a”) and (“polymorphism” or “rs2910164” or “single nucleotide” or “genotype”). We used the Chinese search terms for the Wanfang and CNKI searches are as follows: “diabetes mellitus” or “DM”) and “MCP-1” (“miR-146a” or “microRNA-146a” or “miRNA-146a”) and “polymorphism.”

#### Inclusion and exclusion criteria

The studies are considered acceptable if they (1) are observational studies, including cohort and case–control studies; (2) have at least two comparison groups (T2DM group and control group); and (3) provide the frequencies of genotypes for the rs2910164 polymorphism in T2DM group and control group. The studies are excluded if they are (1) repetitive studies; (2) animal studies; (3) without available data; and (4) lack of control group.

#### Data extraction and quality evaluation

The two investigators (CWW and WLY) independently screened the literature according to the inclusion and exclusion criteria. Any inconsistencies were resolved by the group members. The extracted information mainly included the year of publication, the first author’s name, country (region) of study, ethnicity, sample size, HWE, and genotype distribution of T2DM group and control group. According to the Newcastle–Ottawa Scale (NOS) (33), the quality of the literature was evaluated independently by two system evaluators who discussed and decided on any uncertainties (ZL and TX). Each article is rated on a scale of 0–9, of which 6–9 points suggest high-quality article.

#### Statistical analysis

This study was analyzed with Stata version 12.0 software. First, we used *χ* ^2^ test to assess whether the frequency of rs2910164 polymorphism in control group conformed to the HWE. *OR* and 95% CI were calculated to evaluate the association between rs2910164 polymorphism and the risk of T2DM. If statistical heterogeneity (*P*_Q_ > 0.10 and *I* ^2^ < 50%) was not found among studies, the fixed effects (FE) model was used to calculate the *OR* and 95% CIs, otherwise, the random effects (RE) model was used (34). Publication bias was used by funnel plots and Egger’s test.

## Results

### Case–control study

#### Baseline characteristics

Clinical and demographic information for the T2DM group and control group is shown in **Table 1**. There were no significant differences between T2DM patients and controls in the age and gender (*P* = 0.266 and 0.990, respectively). Compared with estimates in controls, the BMI, FPG, and WHR were higher in the T2DM group (*t*/*Z* = 6.230, 21.726, and 6.913, respectively; all *P* < 0.001).

**Table 1:** General characteristics of the study population.

Variable	Control (*n* = 596)	T2DM (*n* = 574)	*t*/*χ* ^2^/*Z*	*P*
Age (years), mean ± SD	70.88 ± 5.83	70.50 ± 5.86	1.114	0.266
Age, *n* (%)				
< 65	60 (10.1)	69 (12.0)	1.138	0.286
≥ 65	536 (89.9)	505 (88.0)		
Gender, *n* (%)			0.000	0.990
Male	265 (44.5)	255 (44.4)		
Female	331 (55.5)	319 (55.6)		
BMI (kg/m^2^), mean ± SD	23.85 ± 3.51	25.15 ± 3.64	6.230	< 0.001
BMI, *n* (%)				
< 18.5	34 (5.7)	17 (3.0)	21.518	< 0.001
18.5-	278 (46.6)	209 (36.4)		
≥24	284 (47.7)	348 (60.6)		
FPG (mmol/L), median (*P*_25_, *P*_75_)	5.55 (5.07, 6.00)	7.53 (6.55, 9.03)	21.726	< 0.001
WHR, mean ± SD	0.91 ± 0.07	0.94 ± 0.07	6.913	< 0.001

#### Association of rs2910164 loci with T2DM

The rs2910164 CC, CG, and GG genotype frequencies were 36.6%, 47.1%, and 16.3% in the control group and 31.9%, 50.7%, and 17.4% in the T2DM group. The genotype distributions rs2910164 in the control group and in the T2DM group conformed to HWE (*P* = 0.687 and 0.394, respectively). There were no significant differences in genotype frequencies between the T2DM and control group (*P* > 0.05). After adjustment for age, gender, BMI, and WHR, there were no significant differences in genotype frequencies between the T2DM and control group in all genetic models (*P* > 0.05). The specific results are shown in **Table 2**.

**Table 2:** The relationship of miR-146a rs2910164 polymorphism with T2DM.

Models	Group	Control (*n* = 596), *n* (%)	T2DM (*n* = 574), *n* (%)	Crude *OR*(95% CI)	*P*	Adjusted *OR* ^a^ (95% CI)	*P*
genotype							
	CC	218 (36.6)	183 (31.9)	1.00 (reference)		1.00 (reference)	
	CG	281 (47.1)	291 (50.7)	0.81 (0.63–1.05)	0.108	0.82 (0.63–1.06)	0.125
	GG	97 (16.3)	100 (17.4)	0.81 (0.58–1.15)	0.238	0.82 (0.58–1.17)	0.273
recessive model							
	CC	218 (36.6)	183 (31.9)	1.00 (reference)		1.00 (reference)	
	CG + GG	378 (63.4)	391 (68.1)	0.81 (0.64–1.03)	0.091	0.82 (0.64–1.05)	0.109
dominant model							
	GG	97 (16.3)	100 (17.4)	1.00 (reference)		1.00 (reference)	
	CC + CG	499 (83.7)	474 (82.6)	1.09 (0.80–1.47)	0.600	1.08 (0.79–1.47)	0.640
homozygote							
	CC	218 (36.6)	183 (31.9)	1.00 (reference)		1.00 (reference)	
	GG	97 (16.3)	100 (17.4)	0.81 (0.58–1.15)	0.238	0.83 (0.58–1.18)	0.305
heterozygote							
	CG	281 (47.1)	291 (50.7)	1.00 (reference)		1.00 (reference)	
	GG	97 (16.3)	100 (17.4)	1.01 (0.73–1.34)	0.978	1.01 (0.72–1.40)	0.969

a Adjusted for age, gender, BMI, and WHR.

#### Subgroup analysis of the association of rs2910164 loci with T2DM

In the subgroup of individuals ≥ 65 years old, compared with the rs2910164 CC genotype, the CG + GG genotype was associated with a decreased risk of T2DM (*OR* = 0.75; 95% CI: 0.58–0.98; *P*_adjusted_ = 0.032). Similarly, in the BMI < 18.5 group, compared with the CC genotype, CG + GG genotype was associated with a decreased risk of T2DM (*OR* = 0.16; 95% CI: 0.03–0.89; *P*_adjusted_ = 0.037). In other subgroups, no associations between genotype and susceptibility were found (**Table 3**).

**Table 3:** Stratified analyses between miR-146a rs2910164 polymorphism and T2DM risk by gender, age, and BMI.

Variable	Case/Control			*OR* (95% CI); *P*			
	CC	CG	GG	CC + CG vs. GG (dominant)	CG + GG vs. CC (recessive)	GG vs. CC(homozygote)	GG vs. CG(heterozygote)
Gender							
Male	85/97	127/124	43/44	1.02 (0.64-1.62); *P* = 0.937	0.87 (0.60-1.24); *P* = 0.435	0.90 (0.54-1.50); *P* = 0.676	1.05 (0.64-1.71); *P* = 0.851
Female	98/121	164/157	57/53	1.14 (0.76-1.72); *P* = 0.528	0.77 (0.56-1.06); *P* = 0.116	0.75 (0.48-1.19); *P* = 0.226	0.97 (0.63-1.50); *P* = 0.895
Gender ^a^							
Male	85/97	127/124	43/44	0.97 (0.61-1.56); *P* = 0.914	0.89 (0.61-1.29); *P* = 0.527	0.96 (0.56-1.65); *P* = 0.883	1.08 (0.66-1.77); *P* = 0.767
Female	98/121	164/157	57/53	1.17 (0.77-1.77);*P* = 0.474	0.76 (0.55-1.06); *P* = 0.106	0.75 (0.47-1.21); *P* = 0.240	0.95 (0.61-1.48); *P* = 0.830
Age							
< 65	23/15	32/35	14/10	1.27 (0.52-3.12); *P* = 0.598	1.50 (0.70-3.24); *P* = 0.302	1.10 (0.39-3.10); *P* = 0.864	0.65 (0.25-1.68); *P* = 0.376
≥ 65	160/203	259/246	86/87	1.06 (0.76-1.47); *P* = 0.729	**0.76 (0.59-0.98); *P* = 0.036**	0.80 (0.56-1.15); *P* = 0.221	1.07 (0.75-1.50); *P* = 0.720
Age ^b^							
< 65	23/15	32/35	14/10	1.48 (0.58-3.77); *P* = 0.415	1.70 (0.76-3.79); *P* = 0.199	1.11 (0.34-3.66); *P* = 0.861	0.57 (0.21-1.53); *P* = 0.261
≥ 65	160/203	259/246	86/87	1.04 (0.74-1.45); *P* = 0.836	**0.75 (0.58-0.98); *P* = 0.032**	0.81 (0.56-1.17); *P* = 0.257	1.09 (0.77-1.56); *P* = 0.618
BMI							
< 18.5	2/14	12/14	3/6	1.00 (0.22-4.61); *P* = 1.000	**0.19 (0.04-0.97); *P* = 0.046**	0.29 (0.04-2.17); *P* = 0.226	1.71 (0.35-8.37); *P* = 0.505
18.5-	72/104	100/129	37/45	1.11 (0.69-1.80); *P* = 0.658	0.88 (0.60-1.28); *P* = 0.501	0.84 (0.50-1.43); *P* = 0.524	0.94 (0.57-1.57); *P* = 0.820
≥ 24	109/100	179/138	60/46	1.08 (0.71-1.64); *P* = 0.727	0.84 (0.60-1.17); *P* = 0.301	0.84 (0.52-1.34); *P* = 0.454	0.99 (0.64-1.55); *P* = 0.980
BMI ^c^							
< 18.5	2/14	12/14	3/6	1.07 (0.22-5.29); *P* = 0.934	**0.16 (0.03-0.89); *P* = 0.037**	0.16 (0.01-2.54); *P* = 0.193	1.89 (0.35-10.22); *P* = 0.461
18.5-	72/104	100/129	37/45	1.04 (0.64-1.70); *P* = 0.870	0.90 (0.62-1.32); *P* = 0.604	0.92 (0.53-1.60); *P* = 0.779	0.99 (0.59-1.66); *P* = 0.966
≥ 24	109/100	179/138	60/46	1.09 (0.71-1.67); *P* = 0.696	0.83 (0.59-1.16); *P* = 0.263	0.83 (0.51-1.34); *P* = 0.437	1.00 (0.64-1.56); *P* = 0.987

Bold values are statistically significant (P < 0.05). a Adjusted for age, BMI, and WHR; b Adjusted for gender, BMI, and WHR; c Adjusted for age, gender, and WHR.

### Meta-analysis

#### Literature search results and quality evaluation

**Figure 1:**
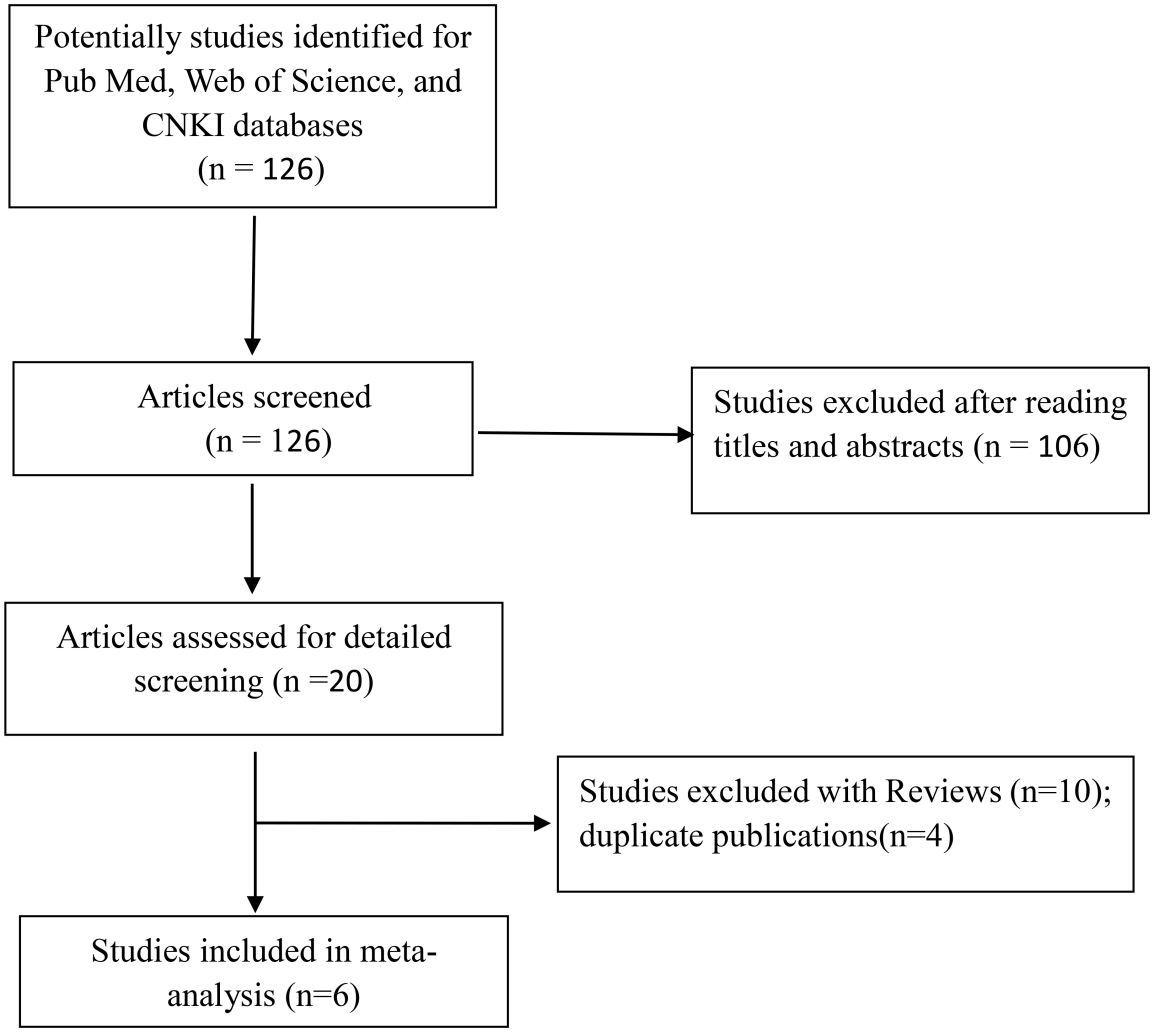
A flow chart of meta-analysis.

According to the search strategy, a total of 126 studies were obtained from the above database, including 68 in English and 58 in Chinese. After reading the title, abstract, and full text, 120 articles were excluded (**Figure 1**). Finally, six articles were included, containing 2,381 T2DM patients and 3,404 controls. Among six included studies, two studies involved Caucasians (one study in Iran and one study in Italy) and four studies involved Asians. The NOS scores of the selected literature were more than 6, suggesting that they were high-quality studies (**Table 4**).

**Table 4:** Characteristics of included studies.

FirstAuthor	Year	Country	Ethnicity	Sample size ^a^	T2DM ^b^	Control ^c^	HWE	NOS score
Ciccacci et al.	2013	Italy	Caucasian	153/181	14/49/90	13/67/101	0.681	8
Wang et al.	2015	China (Heilongjiang)	Asian	995/967	313/506/176	322/477/ 168	0.704	8
Huang et al.	2015	China (Guangdong)	Asian	45/672	12/24/9	225/324/ 123	0.737	8
Li et al.	2015	China (Yunan)	Asian	738/610	364/296/78	236/270/ 104	0.078	7
Alipoor et al.	2016	Iran	Caucasian	183/192	29/62/92	15/65/112	0.208	7
Huang et al.	2021	China (Fujian)	Asian	497/782	187/258/52	308/349/125	0.119	8

Type 2 diabetes mellitus (T2DM), Hardy–Weinberg equilibrium (HWE), Newcastle–Ottawa scale (NOS).

a The total number in the T2DM and control group, respectively.

b The number of CC, CG, and GG genotypes in T2DM group.

c The number of CC, CG, and GG genotypes in control group.

#### The association of miR-146a rs2910164 polymorphism with T2DM risk

In overall analysis, significant heterogeneity was detected (CC + CG vs. GG: *P_Q_* = 0.011, *I* ^2^ = 66.4%; CC vs. CG + GG: *P_Q_* = 0.001, *I* ^2^ = 76.0%; CC vs. GG: *P_Q_* = 0.002, *I* ^2^ = 72.8%; CG vs. GG: *P_Q_* = 0.063, *I* ^2^ = 52.1%; C vs. G: *P_Q_ =* 0.001, *I* ^2^ = 77.3%) (**Table 5**). No significant association between miR-146a rs2910164 polymorphism and T2DM was observed in all genetic models under RE models (CC + CG vs. GG: *OR* = 1.09, 95% CI: 0.83–1.42, *P* = 0.533; CC vs.CG + GG: OR = 1.15, 95% CI: 0.87–1.53, *P* = 0.337; CC vs. GG: *OR* = 1.38, 95% CI: 0.95–1.99, *P* = 0.088; CG vs. GG: *OR* = 1.20, 95% CI: 0.95–1.51, *P* = 0.128; C vs. G: *OR* = 1.13, 95% CI: 0.93–1.35, *P* = 0.213) (**Table 5**).

**Table 5:** The meta-analysis of the association between T2DM and miR-146a rs2910164.

Genetic variant	Ethnicity	No.	Test of heterogeneity		Test of association (FE model)		Test of association (RE model)		P*
			*I* ^2^	*P*	OR (95% CI)	*P*	OR (95% CI)	*P*	
CC + CG vs. GG (dominant)	Overall	6	66.4	0.011	1.10 (0.95-1.26)	0.206	1.09 (0.83-1.42)	0.533	0.932
	Asian	4	76.4	0.005	1.09 (0.93-1.28)	0.305	1.07 (0.74-1.55)	0.714	
	Caucasian	2	54.1	0.140	1.12 (0.83-1.51)	0.445	1.11 (0.72-1.73)	0.631	
CC vs. CG + GG (recessive)	Overall	6	76.0	0.001	1.10 (0.98-1.23)	0.106	1.15 (0.87-1.53)	0.337	0.602
	Asian	4	81.3	0.001	1.07 (0.95-1.20)	0.268	1.04 (0.76-1.41)	0.808	
	Caucasian	2	4.0	0.307	**1.79 (1.08-2.96)**	**0.023**	**1.78 (1.06-2.98)**	**0.029**	
CC vs.GG (homozygote)	Overall	6	72.8	0.002	1.32 (1.12-1.56)	0.001	1.38 (0.95-1.99)	0.088	0.712
	Asian	4	80.5	0.001	1.27 (1.07-1.52)	0.007	1.27 (0.81-1.98)	0.292	
	Caucasian	2	34.7	0.216	**1.79 (1.07-3.00)**	**0.027**	1.75 (0.91-3.35)	0.092	
CG vs. GG (heterozygote)	Overall	6	52.1	0.063	1.20 (1.03-1.39)	0.018	1.20 (0.95-1.51)	0.128	0.993
	Asian	4	60.3	0.056	1.26 (1.07-1.50)	0.007	1.31 (0.97-1.76)	0.079	
	Caucasian	2	10.6	0.290	0.98 (0.71-1.36)	0.921	0.98 (0.70-1.36)	0.923	
C vs. G (allele)	Overall	6	77.3	0.001	1.21 (1.03-1.21)	0.006	1.13 (0.93-1.35)	0.213	0.883
	Asian	4	83.6	0.000	1.10 (1.01-1.20)	0.021	1.09 (0.87-1.37)	0.441	
	Caucasian	2	67.4	0.080	1.23 (0.97-1.55)	0.089	1.21 (0.80-1.83)	0.369	

Number of studies (No.), type 2 diabetes mellitus (T2DM), random effects (RE), fixed effects (FE). *: Test for publication bias (Egger’s test).

**Figure 2:**
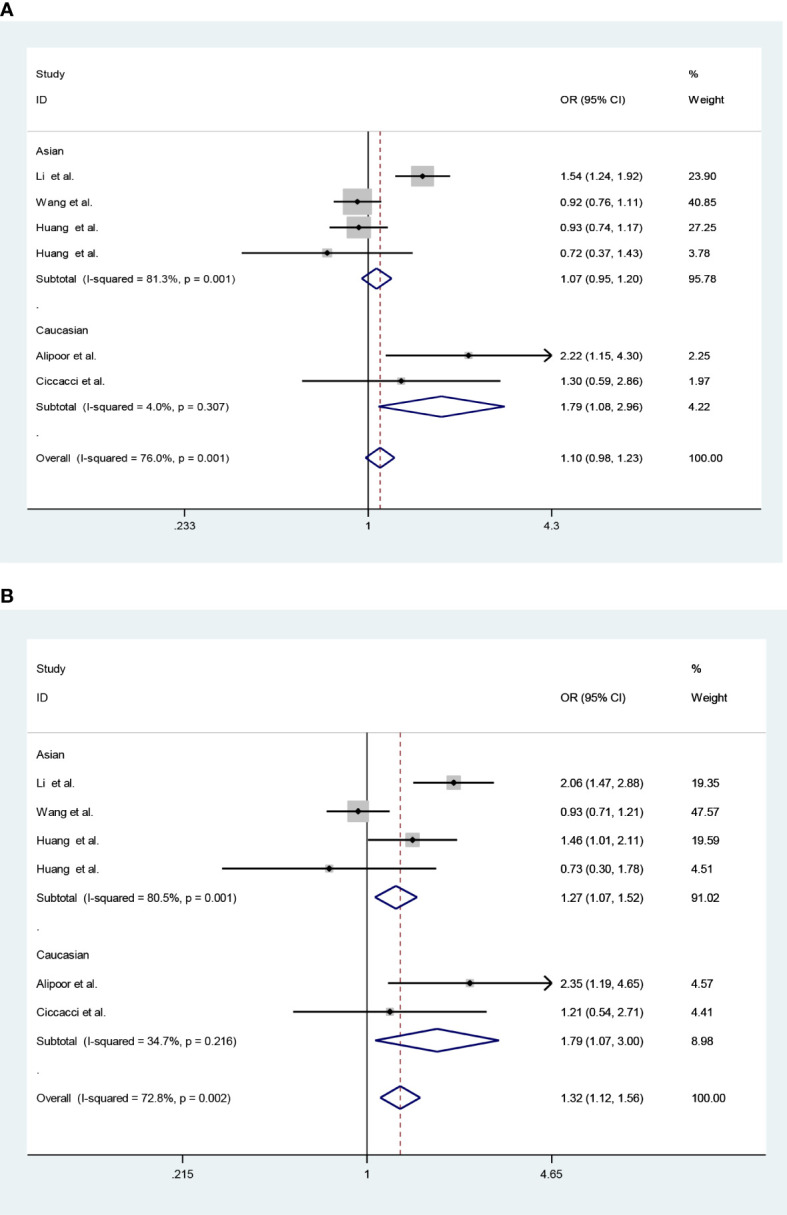
Forest plots (fixed model) for the association of the miR-146a rs2910164 polymorphism with T2DM risk **(A)** recessive model, CC vs. CG + GG; **(B)** homozygote model, CC vs. GG).

Considering heterogeneity, we performed a subgroup analysis of ethnicity. Subgroup analysis showed that there was a significant association in recessive model (CC vs. CG + GG: *OR* = 1.79; 95% CI: 1.08–2.96; *P_Q_* = 0.307, *I* ^2^ = 4.0%) and homozygote model (CC vs. GG: *OR* = 1.79; 95% CI: 1.07–3.00; *P_Q_* = 0.216, *I* ^2^ = 34.7%) under FE models in Caucasians (**Table 5**; **Figure 2**).

#### Sensitivity analysis and publication bias

**Figure 3:**
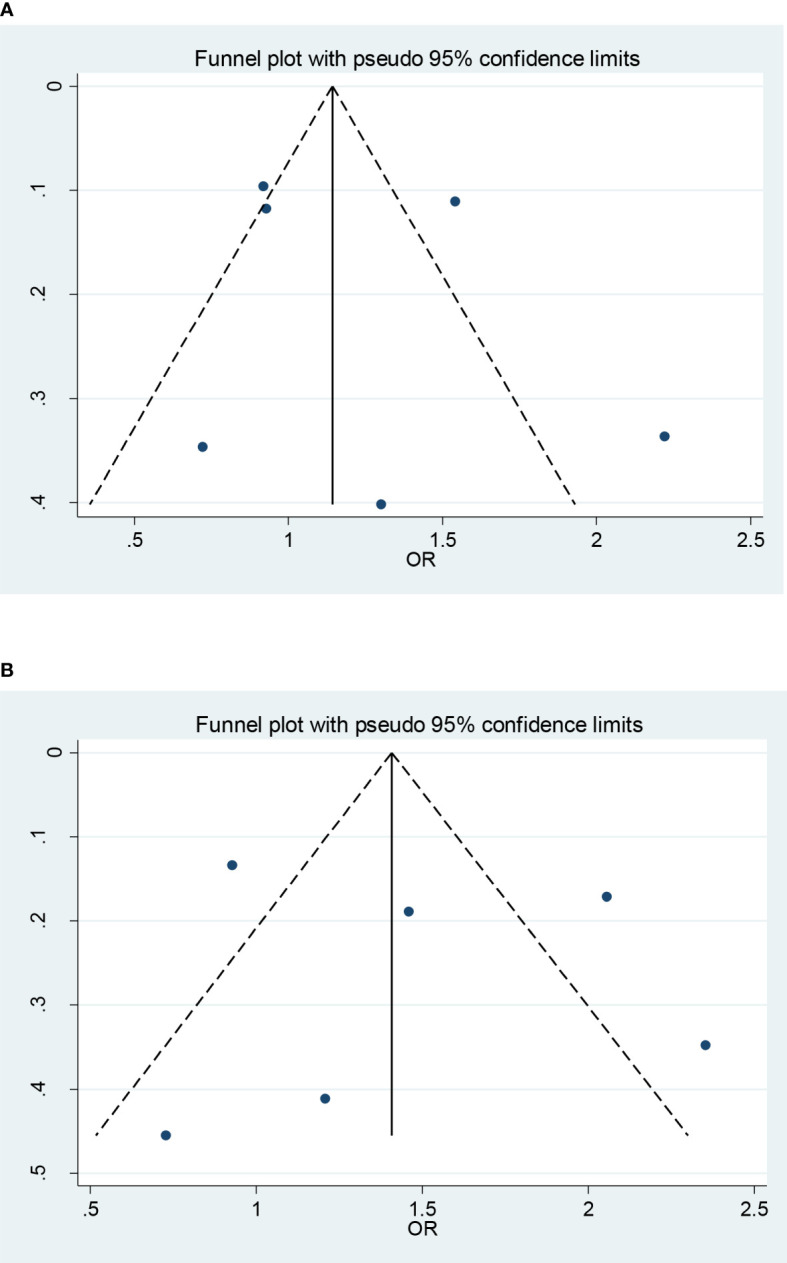
Funnel plots (fixed model) for the association of the miR-146a rs2910164 polymorphism with T2DM risk **(A)** recessive model, CC vs. CG + GG; **(B)** homozygote model, CC vs. GG).

Sensitivity analysis by using the leave-one-out method yielded a similar result compared with the non-sensitivity analysis, indicating our findings are trustworthy (data not shown). Funnel plot and Egger’s test showed that there was no publication bias in each genetic model (CC + CG vs. GG: *P* = 0.932; CC vs. CG + GG: *P* = 0.602; CC vs. GG: *P* = 0.712; CG vs. GG:*P* = 0.993; C vs. G:*P* = 0.883; **Figure 3**).

## Discussion

Inflammation and genetic variation are important factors in the pathogenesis of T2DM (5, 35). Jazdzewski K et al. indicated that the rs2910164 C allele in pre–miR-146a could reduce the expression of miR-146a (36). Xie et al. found that there was a negative correlation between the expression of miR-146a and inflammation-related factors (TNF-α and IL-1β) in diabetic mice (37). Population studies have reported that the miR-146a expression levels in peripheral blood mononuclear cells and plasma are significantly decreased in patients with T2DM (38–40). These findings suggest that miR-146a rs2910164 is involved in the occurrence and development of T2DM.

In the current case–control study, no significant association was found between rs2910164 and T2DM risk in the Chinese population, which is consistent with some of the previous studies (21, 24, 25). After adjustment, it is worth noting that CG/GG genotypes were found to be associated with a decreased risk of T2DM in the subgroup of individuals ≥ 65 years old and BMI < 18.5. Gaytán-Pacheco et al. indicated that miR-146a had indirect relation with peroxisome proliferator-activated receptor (PPAR)-γ expression(41), and PPAR-γ participated in adipocyte differentiation and regulation of lipid metabolism (42). In addition, PPAR-γ was a target mediated by miR-146a (43). Moreover, a recently cross-sectional study revealed that there was a significant interaction between miR-146a and BMI associated with FBG (44). Whether the rs2910164 interacts with BMI in T2DM remains to be further explored. In contrast to the results of our case–control study, several studies suggested that the miR-146a rs2910164 polymorphism was associated with T2DM. Alipoor et al. analyzed two SNPs of miRNAs in a study, namely, 183 T2DM patients and 192 non-diabetic subjects, and the results showed that individuals with rs2910164 CC genotype had a higher risk of T2DM than CG + GG genotypes in an Iranian population (*OR* = 2.3, 95% CI: 1.07-4.90, *P* = 0.031) (25). Similar to the study of Alipoor et al., Li et al. reconfirmed that the C allele of rs2910164 in miR-146a increased the risk of developing T2DM in Chinese population (24). The discrepancies between the studies may be due to the possibly small effect size of this genetic polymorphism to T2DM, the small sample size in each study, and population heterogeneity (45).

The meta-analysis approach is an important and reliable method for identifying associations between genetic variants and diseases based on the results of multiple studies to improve statistical power (46). Several recent meta-analyses have reported an association between the miR-146a rs2910164 polymorphism and T2DM risk (47–50). First, the results of these meta-analyses differ. In a meta-analysis (49), the miR-146a rs2910164 polymorphism was not associated with diabetes (including T1DM and T2DM). Another study has indicated that the miR-146a rs2910164 polymorphism is an ethnicity-dependent susceptibility factor for diabetes (including T1DM and T2DM), and the GG genotype is associated with an increased risk for DM (48). These two meta-analyses included patients with T1DM and T2DM. Another two meta-analyses have focused on T2DM populations and also yielded conflicting results (47, 50). Second, redundant studies were not included in the above meta-analyses (23, 26). Therefore, to derive a more precise estimation of associations, we conducted a larger meta-analysis of all available case–control studies.

To the best of our knowledge, this is the first application of a meta-analysis combined with a case–control study to systematically explore the relationship between the rs2910164 polymorphism and T2DM in different populations. Our meta-analysis, based on six studies involving 2,381 patients with T2DM and 3,404 controls, indicated that there is no significant association between the miR-146a rs2910164 polymorphism and T2DM risk in the overall population under all genetic models. Heterogeneity was detected in all models; accordingly, we conducted stratified analyses according to ethnicity. Heterogeneity was not detected under all genetic models in Caucasians but was detected under most genetic models in Asians. The stratified analysis revealed that the CC genotype might be a risk factor for T2DM susceptibility in Caucasians but not in Asians. In line, we found that miR-146a rs2910164 was not related to risk of T2DM in Chinese population in the present case–control study. The results of the present meta-analysis and case–control study indicated that the effect of the miR-146a rs2910164 polymorphism on T2DM might be ethnicity-dependent, consistent with the results of a study by Chen et al. (48). There are several potential explanations for the different roles of the locus among ethnic groups. First, ethnic differences in genotype distributions may affect the association. T2DM exhibits genetic heterogeneity in different populations (51). The C allele frequency of miR-146a rs2910164 is 25.7% in Italy (21), 38.3% in China (26), and 24.8% in Iran (25). Second, this difference may be due to clinical heterogeneity. Differences in gender, age, disease history, disease severity, BMI, and T2DM diagnosis may influence the association between the locus and the diseases (52–54). It has been reported that the G allele for miR-146a rs2910164 in BMI ≥ 24 kg/m^2^ and never drinking subgroups decreases T2DM susceptibility (26). However, most relevant studies do not provide genotype frequencies for each layer (e.g., gender, age, and BMI).

In this study, strict inclusion and exclusion criteria were established for the meta-analysis, and the results were stable and reliable. However, the meta-analysis and case–control study still had several limitations. First, heterogeneity was detected under all genetic models in overall population. After a stratified analysis according to ethnicity, heterogeneity among studies for the Caucasian subgroup was greatly reduced; however, heterogeneity among studies in the Asian subgroup was still high. Heterogeneity may be affected by gender, age, smoking, BMI, and other factors. Most of the included studies did not analyze the association between miR-146a rs2910164 and T2DM according to these factors, making it difficult to explore the source of heterogeneity. Second, in our meta-analysis, only studies published in Chinese and English were included, which may lead to a language bias. Third, the case–control study did not account for behavioral lifestyle variables related to T2DM, such as smoking, drinking, and exercise, and we were unable to perform subgroup analyses based on these factors. Fourth, the case–control study did not focus on T2DM complications, which may limit the clinical value miR-146a rs2910164 for assessing prognosis.

In summary, the results of our meta-analysis and case–control study demonstrate that the miR-146a rs2910164 polymorphism might have ethnicity-dependent effects in T2DM and may be related to T2DM susceptibility in Caucasians. However, larger case–control and well-designed observational studies are required to verify the observed association in Caucasians.

## Data availability statement

The data presented in the study are publicly available. This data can be found here: https://www.ncbi.nlm.nih.gov/SNP/snp_viewTable.cgi?handle=WMC.

## Ethics statement

The studies involving human participants were reviewed and approved by the Scientific Research and New Technology Ethics Committee of Yijishan Hospital, Wannan Medical College. The patients/participants provided their written informed consent to participate in this study.

## Author contributions

CGM, JYL, and CWW contributed to the study design and acquisition of data. CWW, TX, and WLY analyzed and interpreted the data. CWW and ZL wrote the original manuscript. WLY, ZL, and CGM revised the manuscript. All authors contributed to the article and approved the submitted version.

## Acknowledgments

This project was supported by grants from the National Natural Science Foundation of China (82003546), excellent and top-notch talent cultivation project in colleges and universities in Anhui Province(gxgnfx2022039), the Anhui Natural Science Foundation (1808085QH252; 1808085MH297), and talents Program for Academic Leaders and Reserve Candidates of Wannan Medical College (No. School Administration Letter [2021] No. 46). We would like to thank the editors of this manuscript and Editage (www.editage.cn) for English language editing.

## Conflict of interest

The authors declare that the research was conducted in the absence of any commercial or financial relationships that could be construed as a potential conflict of interest.

## Publisher's note

All claims expressed in this article are solely those of the authors and do not necessarily represent those of their affiliated organizations, or those of the publisher, the editors and the reviewers. Any product that may be evaluated in this article, or claim that may be made by its manufacturer, is not guaranteed or endorsed by the publisher.
